# Comparative effects of different types of exercise interventions on addictive craving, negative affect, and cognitive function in substance use disorder: a substance-subgroup stratified network meta-analysis

**DOI:** 10.3389/fpsyt.2026.1899323

**Published:** 2026-07-30

**Authors:** Haixin Li, Baoxia Chen, Shuting Shao, Junfei Zhang, Zhenguo Shi

**Affiliations:** 1 School of Physical Education, Shandong University, Jinan, China; 2 School of Nursing, Jilin University, Changchun, China; 3 School of Philosophy and Social Development, Shandong University, Jinan, China

**Keywords:** addictive craving, aerobic exercise, exercise intervention, network meta-analysis, substance use disorder

## Abstract

**Objective:**

Recovery in patients with substance use disorder (SUD) involves improvement across addictive craving, negative affect, and cognitive function, with differential responses across neuropharmacological subgroups. This study analyzed the effects of different exercise modalities to provide evidence for symptomatic management in SUD patients.

**Methods:**

Seven databases (PubMed, Web of Science, Cochrane Library, EBSCO, Scopus, Google Scholar, and CNKI) were systematically searched. Risk of bias was assessed using the Cochrane RoB 2 tool, and a frequentist consistency model was fitted in Stata 19. The standardized mean difference (SMD) with 95% confidence interval (CI) served as the effect size; the surface under the cumulative ranking curve (SUCRA) quantified relative efficacy.

**Results:**

Forty-four RCTs (57 independent comparisons, 3,198 patients) were included. For addictive craving, multicomponent exercise (ME; SUCRA = 0.882) and aerobic exercise (AE; SUCRA = 0.749) ranked highest and were statistically significant versus control (CON). For negative affect, AE (SUCRA = 0.880) and ME (SUCRA = 0.697) ranked highest with statistical significance. For cognitive function, AE ranked highest (SUCRA = 0.770), though no intervention reached significance versus CON. Subgroup analysis showed ME, mind–body exercise (MBE), and AE ranked high and were significant for craving in stimulant use disorder (StUD); AE ranked high and was significant in non-stimulant use disorder (Non-StUD).

**Conclusion:**

Exercise effects in SUD patients are outcome-specific and substance subtype-dependent. ME and AE showed clear benefits for addictive craving; AE and ME positively affected negative affect; AE demonstrated a stable effect on cognitive function. ME, MBE, and AE may be more effective for craving in StUD, whereas AE and resistance exercise (RSE) may be preferable in Non-StUD. These findings may inform symptom-targeted and stratified exercise-based rehabilitation for SUD patients.

**Systematic review registration:**

https://www.crd.york.ac.uk/prospero/, identifier CRD420261388220.

## 1 Introduction

Substance use disorder (SUD) is a category of chronic mental health conditions characterized at its core by persistent substance use, intense craving, diminished control, and repeated relapse (1). In World Health Statistics 2024, the World Health Organization identified alcohol and drug use disorders as one of the key contributors that continue to drive the global burden of avoidable disease (2); in the same year, the World Drug Report released by the United Nations Office on Drugs and Crime further noted an overall upward trend in global illicit drug use and related health problems (3). At the neurobiological level, the onset and maintenance of SUD involve multiple mechanisms, including sensitization of reward circuits, activation of stress and negative-affect circuits, and decline in prefrontal executive control (4), which together form a vicious cycle of “intensified craving–accumulated negative affect–weakened cognitive control–increased relapse risk.” Consequently, evaluating recovery solely on the basis of the frequency of substance use or the duration of abstinence cannot fully capture the cross-symptom recovery process in patients with SUD, and a systematic assessment from a multidimensional outcome perspective is warranted.

Within the SUD recovery process, core addiction symptoms, negative affective symptoms, and cognitive function constitute three key dimensions that are interrelated yet relatively independent, corresponding respectively to functional abnormalities in the “reward–motivation–executive control” neural circuits, and may serve at the mechanistic level as stable anchors for assessing the targeted effects of exercise interventions. Addiction craving symptoms (Outcome 1, O1) center on craving intensity, withdrawal discomfort, the urge to use substances, and severity of dependence, and represent a key variable that reflects the core pathological process of addiction and directly predicts relapse risk (5, 6). Negative affective symptoms (Outcome 2, O2) encompass comorbid affective problems such as depression, anxiety, stress, and negative mood, and are closely related to negative-reinforcement mechanisms and stress-related relapse pathways (4). Cognitive function (Outcome 3, O3) in this review refers mainly to cognitive-control-related domains, including inhibitory control, attentional regulation, modulation of impulsivity, executive function, and decision-making, and constitutes the cognitive basis on which patients with SUD rely to resist triggers, maintain abstinence, and restore social functioning (7).

As a safe, low-cost, and scalable adjunctive strategy, exercise has been incorporated into the rehabilitation management of patients with SUD (8–10). Different types of exercise may influence craving, affect, and cognitive function in patients with SUD through different physiological and psychological pathways. Aerobic exercise (AE), centered on cardiorespiratory endurance training, may act to improve overall physical and mental status by modulating monoaminergic and endogenous opioid neurotransmitter systems (8). Yoga- or mindfulness-based exercise (YME), centered on yoga postures and breath regulation, emphasizes attentional regulation and emotional stabilization. Mind–body exercise (MBE), represented by traditional Eastern mind–body practices such as Tai Chi, Qigong, and Baduanjin, regulates autonomic balance and affect–cognition coupling through movement control, coordinated breathing, and mind–body integration. Resistance exercise (RSE), centered on muscular strength and resistance stimulation, may exert its effects by enhancing muscle strength, self-efficacy, and a sense of bodily control (11, 12). Multicomponent exercise (ME), by integrating multiple training stimuli, may act simultaneously across several outcomes (13).

Although a number of studies have recently examined the role of exercise interventions in SUD recovery (13–16), the existing evidence has largely been analyzed from the perspective of whether exercise is effective overall, and has less often jointly considered the correspondence among exercise type, symptom outcome, and substance subgroup. The neuropsychological mechanisms underlying addictive craving, depression and anxiety, sleep disturbance, and impaired cognitive control are not entirely consistent; moreover, different exercise modalities may show differentiated effects on specific symptom targets because of their distinct training stimuli and pathways of action. Furthermore, the patterns of neuropharmacological impairment caused by different substance types also differ in important ways. Stimulant substances are typically closely associated with overactivation of monoaminergic systems, sensitization of reward pathways, and impaired prefrontal control; non-stimulant substances, by contrast, more often involve abnormalities in μ-opioid receptors, the endogenous cannabinoid system, and motivation–affect regulation pathways (4, 7, 17). This substance-related mechanistic heterogeneity suggests that the response of patients with SUD to exercise interventions may not be entirely uniform, particularly for the addictive craving outcome, which is closely related to reward sensitization and relapse risk, where different substance subgroups may display differentiated exercise-response patterns. Therefore, building on the integration of multiple types of exercise interventions and multidimensional symptom outcomes, the present study aimed to systematically evaluate, from the dual perspectives of exercise type and substance subgroup, the relative effects of exercise interventions on craving, affective symptoms, cognitive function, and related health outcomes in patients with SUD, with the aim of providing more targeted evidence for optimizing exercise prescriptions in SUD recovery.

On this basis, the present study used a network meta-analysis to systematically compare the effects of five types of exercise interventions, namely AE, YME, MBE, RSE, and ME, on addictive craving symptoms (O1), negative affective symptoms (O2), and cognitive function (O3) in patients with SUD. Given the close relationship among addictive craving, reward sensitization, motivational drive, and relapse risk, O1 was further divided, according to neuropharmacological substance category, into two subgroups, namely stimulant use disorder (StUD) and non-stimulant use disorder (Non-StUD), in order to explore the relative efficacy rankings of exercise interventions for addictive craving across substance subgroups. By simultaneously comparing different outcome measures and substance subgroups, this study aimed to provide more targeted evidence-based support for optimizing exercise-based rehabilitation programs in patients with SUD, and to offer exploratory evidence for constructing precision exercise prescriptions based on the matching among “symptom target–substance type–exercise mechanism.”

## 2 Methods

### 2.1 Study design and reporting standards

This study is a systematic review and network meta-analysis. Its design, conduct, and reporting followed the PRISMA 2020 statement (18) and its network meta-analysis extension (PRISMA-NMA) (19), with the methodological framework guided by the Cochrane Handbook for Systematic Reviews of Interventions (20). The study protocol was prospectively registered on the PROSPERO platform (registration number: CRD420261388220).

### 2.2 Search strategy

Seven Chinese and English databases were systematically searched, namely PubMed, Web of Science, Cochrane Library, EBSCO, Scopus, Google Scholar, and China National Knowledge Infrastructure (CNKI), from database inception to January 30, 2026. Taking PubMed as an example, the search string was ((“substance use disorder” OR addiction OR “drug dependence” OR “substance dependence”) AND (exercise OR “aerobic exercise” OR yoga OR mindfulness OR “mind-body exercise” OR “resistance exercise” OR “strength training” OR “multicomponent exercise”) AND (craving OR withdrawal OR depression OR anxiety OR “cognitive control” OR “executive function” OR impulsivity) AND (“randomized controlled trial” OR trial OR intervention)), with the final search string iteratively refined during formal implementation. The search was conducted independently by two researchers, and the reference lists of experimental studies and relevant reviews were manually screened to identify additional potentially eligible studies. During revision, the search results and potentially relevant records were re-checked using the same prespecified PICOS criteria, with particular attention to randomized exercise-based intervention studies that may not have been captured or may have been excluded during full-text screening.

### 2.3 Inclusion and exclusion criteria

Inclusion and exclusion criteria were established according to the Population, Intervention, Comparator, Outcome, and Study design (PICOS) framework. Inclusion criteria were as follows. (i) Participants (P) were patients with SUD who met DSM-5/DSM-5-TR, ICD-10/ICD-11, or other recognized diagnostic criteria; substance types could include alcohol, opioids, methamphetamine, cocaine, tobacco, cannabis, or other psychoactive substances, with the specific substance type determined by the original study report. (ii) The intervention (I) was a structured exercise intervention that explicitly reported basic parameters such as exercise type, intervention period, training frequency, session duration, and intensity, and that could be classified into one of the five categories of AE, YME, MBE, RSE, or ME. (iii) The comparator (C) was any non-exercise control, uniformly coded as a Control (CON) node for inclusion in the network analysis, so as to ensure the statistical basis of the network. (iv) The outcome (O) was at least one of the three categories of continuous outcome measures: addictive craving symptoms, negative affective symptoms, and cognitive function. (v) The study type (S) was a randomized controlled trial.

Exclusion criteria were as follows. (i) Ineligible publication type: reviews, systematic reviews, meta-analyses, study protocols, conference abstracts, dissertations (for which only abstracts were available), editorials, commentaries, animal or *in vitro* studies, case reports, and case series. (ii) Data not retrievable: key continuous outcome data (post-test mean, standard deviation or a convertible dispersion statistic, and sample size) were missing. (iii) Intervention effect could not be isolated: the exercise intervention was implemented jointly with other active interventions such as pharmacotherapy or cognitive behavioral therapy, and the original study did not provide a control design or subgroup data that could isolate the independent effect of exercise. (iv) Duplicate data: when multiple reports existed for the same cohort or dataset, only the version with the largest sample size and the most complete information was retained, and the others were excluded.

### 2.4 Study selection and data extraction

Study selection was performed independently by two researchers. An initial screening was first conducted on the basis of titles and abstracts to exclude studies that clearly did not meet the inclusion criteria; a secondary screening was then performed through full-text reading, and the reason for exclusion was recorded for each excluded study. Disagreements were resolved through discussion, and a third researcher adjudicated when necessary. Potentially relevant studies identified during the re-check were retained only when they clearly met the prespecified eligibility criteria; studies with unclear or non-random allocation, non-extractable outcome data, non-isolatable exercise effects, incompatible comparators, or duplicate data were not included in the quantitative network meta-analysis.

Data extraction was performed independently by the same two researchers using a pre-designed, standardized Excel form, following the procedure recommended in the Cochrane Handbook for Systematic Reviews of Interventions (20). The extracted items included first author, year of publication, country or region, study design, sample size, patient age, sex composition, substance type, diagnostic criteria, intervention type, control type, intervention period, training frequency, session duration, exercise intensity, outcome scales, measurement time points, and the post-intervention mean, standard deviation, and sample size.

### 2.5 Classification of exercise interventions and outcome measures

Exercise interventions were uniformly classified into five categories. This classification was based on the predominant training component reported in the original trials and on clinically relevant distinctions in exercise content; yoga- or mindfulness-based exercise and traditional mind–body exercise may both be viewed as subtypes within the broader psychomotor/mind–body exercise category, but they were retained as separate nodes to reduce within-node heterogeneity and to reflect their different emphases on posture–breathing–awareness training versus coordinated movement–breathing integration. (i) Aerobic exercise, centered on continuous cardiorespiratory endurance training, with typical forms including walking, running, cycling, swimming, and aerobic dance; (ii) Yoga- or mindfulness-based exercise (YME), with yoga postures, breath regulation, and body awareness as core components; (iii) Mind–body exercise (MBE), centered on traditional mind–body practices such as Tai Chi, Qigong, and Baduanjin, which emphasize movement control, coordinated breathing, bodily coordination, and mind–body integration; (iv) Resistance exercise, with muscular strength, muscular endurance, or resistance stimulation as the main training objective; and (v) Multicomponent exercise, an integrated program that simultaneously includes two or more different exercise components within the same intervention period. For mixed studies whose intervention programs contained both yoga/mindfulness elements and Tai Chi/Qigong elements, two researchers independently determined the classification according to the predominant training component, with disagreements adjudicated by a third person. In strict accordance with the recommendations of the Cochrane Handbook for Systematic Reviews of Interventions regarding the extraction of continuous outcome data, the harmonization of effect direction, and pre-specified outcome classification, and in combination with the PICOS framework and clinical relevance established in advance for this study, outcome measures were uniformly classified into three categories: O1, addictive craving symptoms; O2, negative affective symptoms; and O3, cognitive function. Because the included trials used heterogeneous cognitive tasks and scales, O3 was defined at the construct level and primarily covered cognitive-control-related domains such as inhibitory control, attention, working memory, executive function, and reaction-time-based cognitive performance. These subdomains were operationalized through the cognitive tasks reported in the included trials: inhibitory control/response inhibition (Go/No-Go tasks), executive function including interference control (Stroop and other executive-function tasks), working memory (working-memory tasks), attention (attentional-bias tasks and the Attention Network Test), and processing speed (choice reaction-time tasks). For cognitive function outcomes, the extracted metric followed the primary cognitive indicator reported in each original study: accuracy- or error-based indicators were used for tasks primarily assessing response accuracy or inhibition, whereas reaction-time-based indicators were used for tasks primarily assessing response speed or latency; all directions were harmonized so that a higher SMD indicated greater improvement. The integration of scales within each of the three outcome categories was based mainly on construct homology (belonging to the same three neural pathways of reward–withdrawal, negative reinforcement, and prefrontal executive control), the clinical comorbidity of the relevant symptoms in patients with SUD (4), and the cross-instrument comparability of the SMD as a standardized pooling metric; for scales with opposite scoring directions, the effect direction was uniformly reversed.

### 2.6 Principles for substance-subgroup classification

Based on the classification of psychoactive substance categories in DSM-5-TR, this study further conducted a substance-subgroup stratified analysis within the addictive craving symptom (O1) network. O1 was selected as the object of stratification mainly because addictive craving is more directly linked to substance type, withdrawal-induced urges, and relapse risk; the number of studies available for stratification in O2 and O3 was limited, and further stratification might increase the risk of network sparsity and unstable estimation, so these outcomes were interpreted only in the main analysis. Stimulant-type substances (such as methamphetamine and cocaine), whose core pathological features are overactivation of the central dopamine system, sensitization of reward pathways, and impaired prefrontal control, were assigned to the stimulant use disorder subgroup (StUD); the remaining psychoactive substances, whose core pathological features are not centered on dopaminergic overactivation and which clinically often present common features such as low arousal, low motivation, and anhedonia, were assigned to the non-stimulant use disorder subgroup (Non-StUD). In addition, to test the robustness of the classification, this study refitted the Non-StUD network in a sensitivity analysis after excluding studies with unclear descriptions of substance type, and compared whether the main effect directions, statistical significance, and SUCRA rankings changed substantively. Confidence in the network meta-analysis estimates was appraised using the CINeMA framework across six domains (within-study bias, reporting bias, indirectness, imprecision, heterogeneity, and incoherence), summarized into an overall rating of high, moderate, low, or very low.

### 2.7 Risk of bias assessment

The risk of bias of the included studies was assessed using the revised Cochrane risk-of-bias tool, RoB 2 (21). The assessment domains were as follows: (i) bias arising from the randomization process; (ii) bias due to deviations from intended interventions; (iii) bias due to missing outcome data; (iv) bias in measurement of the outcome; and (v) bias in selection of the reported result. Each domain was judged as “low risk,” “some concerns,” or “high risk.” The assessment was performed independently by two researchers, with disagreements resolved through discussion.

### 2.8 Statistical analysis

All network meta-analyses were performed in Stata 19. All three outcomes were continuous variables, and the pooled effect size was expressed as the standardized mean difference (SMD) with its 95% CI. In comparisons with the control group (CON), an SMD > 0 indicated a more favorable effect of the exercise intervention; for scales on which lower scores indicate symptom improvement (such as the BDI, SAS, and SCL-90), the effect direction was uniformly reversed, and the threshold for statistical significance was set at a two-sided P < 0.05. According to the pre-specified outcome classification, independent evidence networks were constructed separately for addictive craving symptoms (O1), negative affective symptoms (O2), and cognitive function (O3), with network nodes comprising the five types of exercise interventions and CON. A random-effects frequentist consistency model was used to integrate direct and indirect evidence (22–24); multi-arm trials were handled using the built-in multi-arm adjustment method of the relevant Stata command, and an augmented covariance matrix was used to correct for the correlation of a shared control group across multiple comparisons. Pairwise comparison results were presented in league tables, and treatment rankings were described using SUCRA. The interpretation of results comprehensively considered the SMD, 95% CI, statistical significance, SUCRA ranking, and network structure, avoiding judgments based on ranking alone. Heterogeneity was assessed using τ² and I², and consistency was evaluated through global and local inconsistency tests. For O1, StUD and Non-StUD subgroups were further defined according to the main substance type used and modeled separately; given the limited subgroup sample sizes, the relevant results were interpreted only as exploratory, and no formal between-subgroup interaction test was performed.

## 3 Results

### 3.1 Study selection process and results

**Figure 1:**
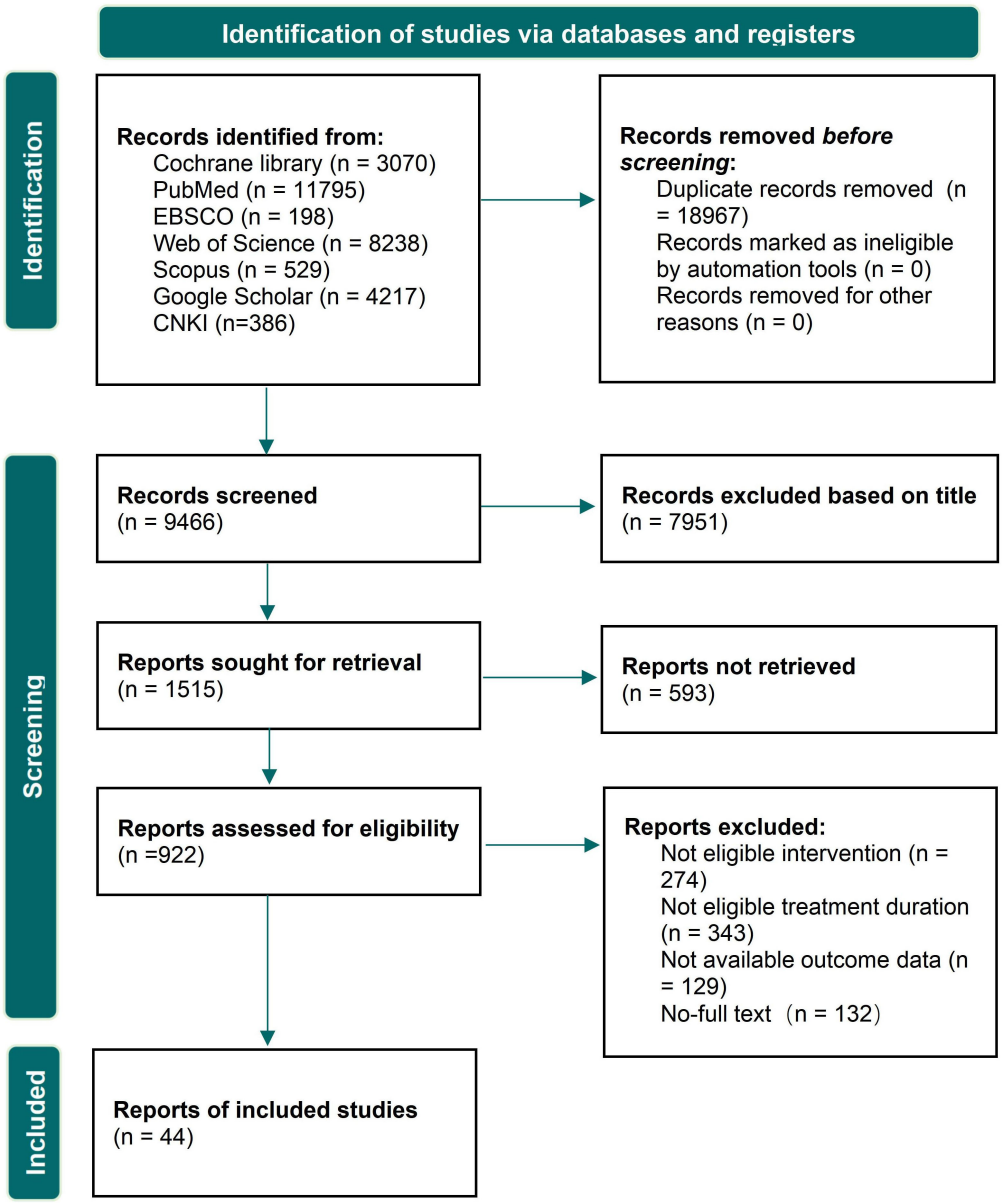
PRISMA flow diagram.

After the pre-specified search strings were used to systematically search the seven Chinese and English databases, 9,466 records remained for initial screening following the removal of duplicates. After studies that clearly did not meet the PICOS framework were excluded on the basis of titles and abstracts, 1,515 records entered the full-text retrieval stage; among these, some were excluded because the full text could not be obtained and did not enter full-text assessment, leaving 922 articles for full-text re-review. Following strict review of the full texts against the predetermined inclusion and exclusion criteria, a total of 44 randomized controlled trials, 57 independent comparisons available for network meta-analysis, and 3,198 patients with SUD that met the quality requirements were finally included. The study selection process strictly followed the PRISMA-NMA requirements (see **Figure 1**).

### 3.2 Basic characteristics of included studies

**Figure 2:**
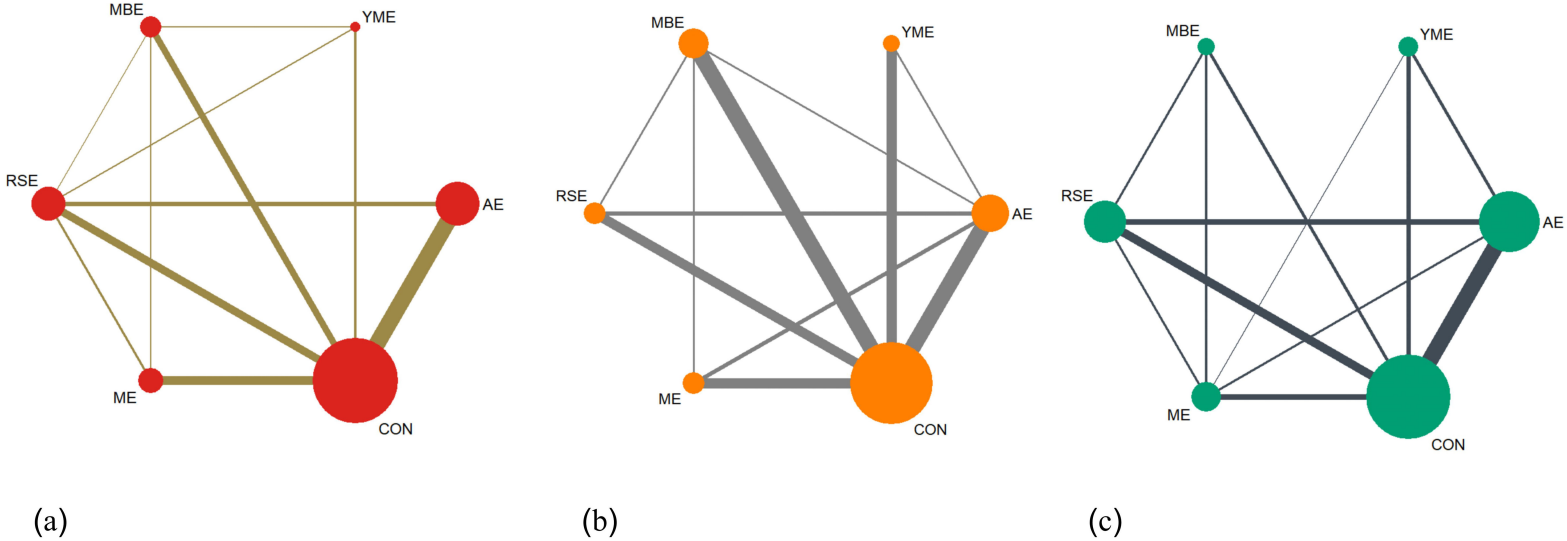
Network diagram of three outcomes. **(a)** addictive craving (O1), **(b)** negative affect (O2), and **(c)** cognitive function (O3).

The 44 articles included in this study covered five types of exercise intervention programs together with control conditions; the basic characteristics of the included studies are presented in **Table 1**. Independent network evidence structures were constructed separately for the three core outcomes; all three networks contained the five exercise intervention nodes (AE, YME, MBE, RSE, ME) and the control node (CON), for a total of six nodes. In the O1 network, direct comparisons between AE and CON were the most numerous in terms of the number of RCTs, reflecting the fact that aerobic exercise, as the earliest type of exercise introduced into SUD rehabilitation, has the deepest evidence base; in the O2 network, dense edges were formed between AE and MBE and CON; in the O3 network, direct comparisons between AE and CON were the densest, followed by the RSE node, while the remaining nodes were informed by comparatively fewer comparisons (see **Table 1**; **Figure 2**).

**Table 1:** Randomized controlled trials included in the article.

Study	Country	Sample size	Age(years)	Exercise intervention category	Intervention period	Frequency	Session duration	Outcome measure(s)
Li et al. (2002) (25)	China	34/26	31.8 ± 6.0	MBE	Low	High	High	HAM-A; withdrawal symptoms; urine morphine
Ussher (2006) (26)	UK	20/20	30.5 ± 8.0	RSE	Low	Low	Low	Desire to smoke; withdrawal symptoms; mood symptoms
Vickers (2009) (27)	USA	30/30	41.4 ± 11.9	ME	Moderate	Low	Low	Smoking abstinence; depression symptoms; exercise behavior
Buchowski (2011) (28)	USA	12/12	24.8 ± 2.9	AE	Low	High	Low	Cannabis craving; cannabis use
Bock (2012) (29)	USA	32/23	43.8 ± 9.4	YME	Moderate	Low	High	Smoking abstinence; anxiety; depression; perceived health
Li D (2013) (30).	China	36/34	30.0 ± 6.7	MBE	High	Moderate	High	Withdrawal symptoms; HRSD depression; physical parameters
Smelson (2013) (31)	USA	51/50	36.0 ± 9.4	MBE	Low	Moderate	Low	Cue-elicited cocaine craving; BDI; STAI
Devi (2014) (32)	India	33/33	32.5 ± 9.9	YME	Moderate	High	High	BDI-II; WHOQOL-BREF
Wang D (2015) (33).	China	24/24	31.5 ± 6.6	AE	Low	Low	Low	MA craving; Go/No-Go inhibitory control; ERP
Agarwal (2015) (34)	USA	12/12	47.0 ± 8.9	YME	Moderate	Low	High	Quality of life; perceived stress; IES; cortisol; DHEA-S
Rawson (2015) (35)	USA	69/66	31.7 ± 6.9	ME	Moderate	Moderate	High	BDI depression; BAI anxiety
Costa (2016) (36)	Brazil	9/9	32.0 ± 4.7	AE	High	Moderate	High	Cardiorespiratory fitness; Stroop cognitive function
De La Garza (2016a) (37)	USA	10/7	43.4 ± 7.4	AE	Moderate	Moderate	Low	Cocaine craving; cocaine use; tobacco craving/use; fitness
De La Garza (2016b) (37)	USA	7/7	45.6 ± 1.6	AE	Moderate	Moderate	Low	Cocaine craving; cocaine use; tobacco craving/use; fitness
Wang (2016a) (38)	China	23/23	33.3 ± 9.1	AE	Low	Low	Moderate	MA craving; Go/No-Go inhibitory control; ERP
Wang (2016b) (38)	China	23/23	32.1 ± 6.3	AE	Low	Low	Moderate	MA craving; Go/No-Go inhibitory control; ERP
Wang (2016c) (38)	China	23/23	34.0 ± 8.9	AE	Low	Low	Moderate	MA craving; Go/No-Go inhibitory control; ERP
Wang D. (2017A) (39)	China	25/25	33.5 ± 7.5	AE	High	Moderate	Moderate	MA craving; inhibitory control; ERP
Wang D. (2017B) (40)	China	32/31	32.2 ± 7.0	AE	High	Moderate	Moderate	Fitness; MA craving; anxiety; depression
Zhang K (2018) (41).	China	68/35	33.3 ± 7.7	AE	High	Moderate	Moderate	Cognitive function; oxidative stress markers
Zhu D. (2018) (42)	China	42/38	33.7 ± 7.1	MBE	High	Moderate	High	Sleep quality; SDS depression; fitness; relapse
Gong (2019) (43)	China	26/26	31.9 ± 6.0	AE	Low	Low	Moderate	Craving; emotional state; neurotransmitters
Lu (2019) (44)	China	36/34	31.5 ± 0.6	ME	High	High	High	SCL-90; SDS; SAS; VAS craving; immune/DA markers
Zhang (2020) (45)	China	38/38	41.1 ± 9.9	MBE	High	High	High	Drug craving; anxiety; depression; physical fitness; QOL
Wang (2020) (46)	China	30/30	32.7 ± 7.2	AE	Low	Low	Moderate	Heroin craving; Go/No-Go inhibitory control; ERP/frequency bands
Liu (2021) (47)	China	165/165	31.3 ± 5.5	AE	Moderate	High	High	Executive function; HRV; cardiopulmonary fitness
Shen (2021) (48)	China	35/37	39.3 ± 10.3	MBE	High	Moderate	High	Executive function; physical fitness
Zhu (2021) (49)	China	42/41	34.6 ± 5.1	AE	High	High	Moderate	Cognitive function; emotions; craving; physical fitness
Ellingsen (2021a) (50)	Norway	36/36	37.3 ± 6.4	AE	Low	Low	Moderate	Drug craving; mood
Ellingsen (2021b) (50)	Norway	36/36	37.3 ± 6.4	RSE	Low	Low	Moderate	Drug craving; mood
Chen (2021a) (51)	China	17/21	32.5 ± 4.8	AE	High	Moderate	Moderate	Cue-induced craving; working memory; inhibitory control
Chen (2021b) (51)	China	19/21	29.6 ± 4.4	AE	High	Moderate	Moderate	Cue-induced craving; working memory; inhibitory control
Brellenthin (2021) (52)	USA	11/10	35.1 ± 10.2	AE	Moderate	Moderate	Moderate	Craving; mood states; depression; anxiety; endocannabinoids
Zhou (2021a) (53)	China	20/19	26.5 ± 4.1	AE	Low	Low	Moderate	Cue-induced MA craving; food reward; fNIRS
Zhou (2021b) (53)	China	19/20	26.5 ± 4.1	AE	Low	Low	Moderate	Cue-induced MA craving; food reward; fNIRS
Xu (2022) (54)	China	30/30	31.3 ± 3.9	AE	High	High	High	QOL-DA; SAS; SDS; PSQI; physical fitness
Welford (2022a) (55)	Sweden	46/45	53.9 ± 10.6	AE	High	Moderate	High	HADS anxiety; HADS depression
Welford (2022b) (55)	Sweden	49/45	54.2 ± 13.4	YME	High	Moderate	High	HADS anxiety; HADS depression
Salem (2022) (56)	USA	69/66	31.7 ± 6.9	ME	Moderate	Moderate	High	MA craving; MA use after discharge
Li H. (2023a) (57)	China	26/25	NR	RSE	Moderate	High	Moderate	Drug craving; sleep quality; cardiovascular and fitness outcomes
Li H. (2023b) (57)	China	25/25	NR	RSE	Moderate	High	Moderate	Drug craving; sleep quality; cardiovascular and fitness outcomes
Liu (2023) (58)	China	23/23	28.5 ± 3.3	AE	Moderate	High	High	Cognitive function; anxiety; depression
Chen (2023) (59)	China	20/20	26.6 ± 4.5	AE	Low	Low	Moderate	Drug craving; prefrontal activation; fNIRS
Zhang (2024) (60)	China	20/24	38.3 ± 9.1	ME	High	Moderate	High	Drug craving; attention bias; physical fitness
Wang M. (2024) (61)	China	48/47	32.5 ± 8.2	MBE	High	High	Moderate	Drug craving
Guo (2024) (62)	China	20/24	39.6 ± 10.2	ME	High	Moderate	Moderate	Drug craving; executive function; physical fitness
Malagodi (2024a) (63)	Brazil	43/43	27.6 ± 5.9	AE	Low	Low	High	Craving; Go/No-Go inhibitory control
Malagodi (2024b) (63)	Brazil	43/43	27.6 ± 5.9	RSE	Low	Low	High	Craving; Go/No-Go inhibitory control
Wang K. (2024a) (64)	China	17/17	NR	AE	High	Moderate	Moderate	Sleep quality; drug craving; EEG prefrontal alpha
Wang K. (2024b) (64)	China	17/17	NR	ME	High	Moderate	Moderate	Sleep quality; drug craving; EEG prefrontal alpha
Yang (2025a) (65)	China	29/29	25.5 ± 4.2	MBE	High	High	Moderate	Relapse tendency; sleep quality; choice reaction time; physical fitness
Yang (2025b) (65)	China	25/25	25.5 ± 4.2	AE	High	High	Moderate	Relapse tendency; sleep quality; choice reaction time; physical fitness
Yang (2025c) (65)	China	26/26	25.5 ± 4.2	ME	High	High	Moderate	Relapse tendency; sleep quality; choice reaction time; physical fitness
Jin (2025a) (66)	China	35/35	30.2 ± 5.5	AE	Low	Low	Moderate	Craving; emotional state; attentional bias; working memory; inhibitory control
Jin (2025b) (66)	China	35/35	30.2 ± 5.5	RSE	Low	Low	Moderate	Craving; emotional state; attentional bias; working memory; inhibitory control
Li (2025) (67)	China	32/32	32.8 ± 5.8	AE	Low	Low	Moderate	Craving; attention network function
Goutham (2026) (68)	India	30/29	26.3 ± 3.3	YME	Low	High	Moderate	COWS withdrawal stabilization; HRV; anxiety; sleep latency; pain

The Low/Moderate/High categories were coded according to exercise-training principles and the dose descriptions reported in the original trials. Intervention period was classified according to total intervention duration, frequency according to sessions per week, session duration according to minutes per session, and intensity according to reported intensity indicators, such as %HRmax, HRR, VO_2_max, METs, RPE, or the original authors’ descriptive intensity level where objective indicators were unavailable.

### 3.3 Quality assessment of included studies

**Figure 3:**
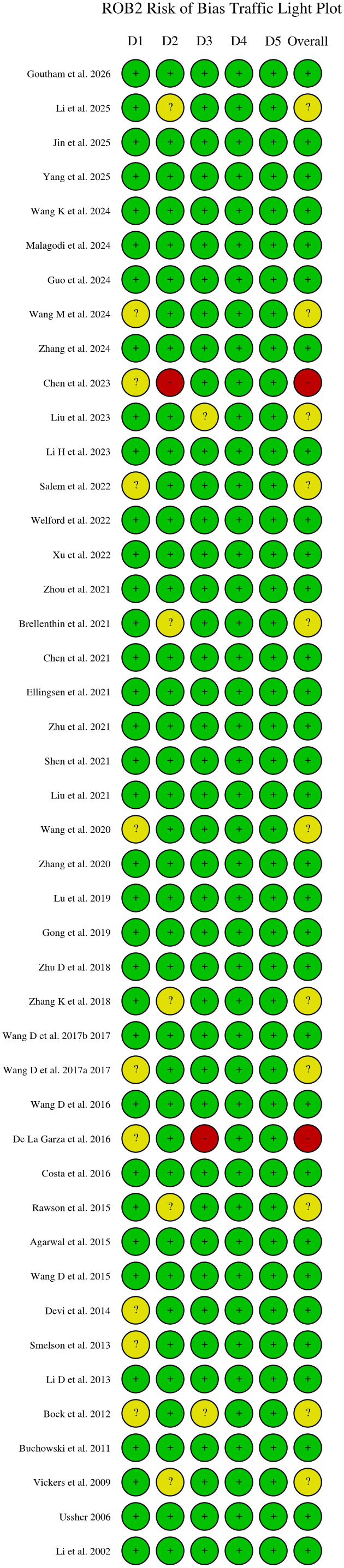
Risk of bias assessment for included studies.

**Figure 4:**
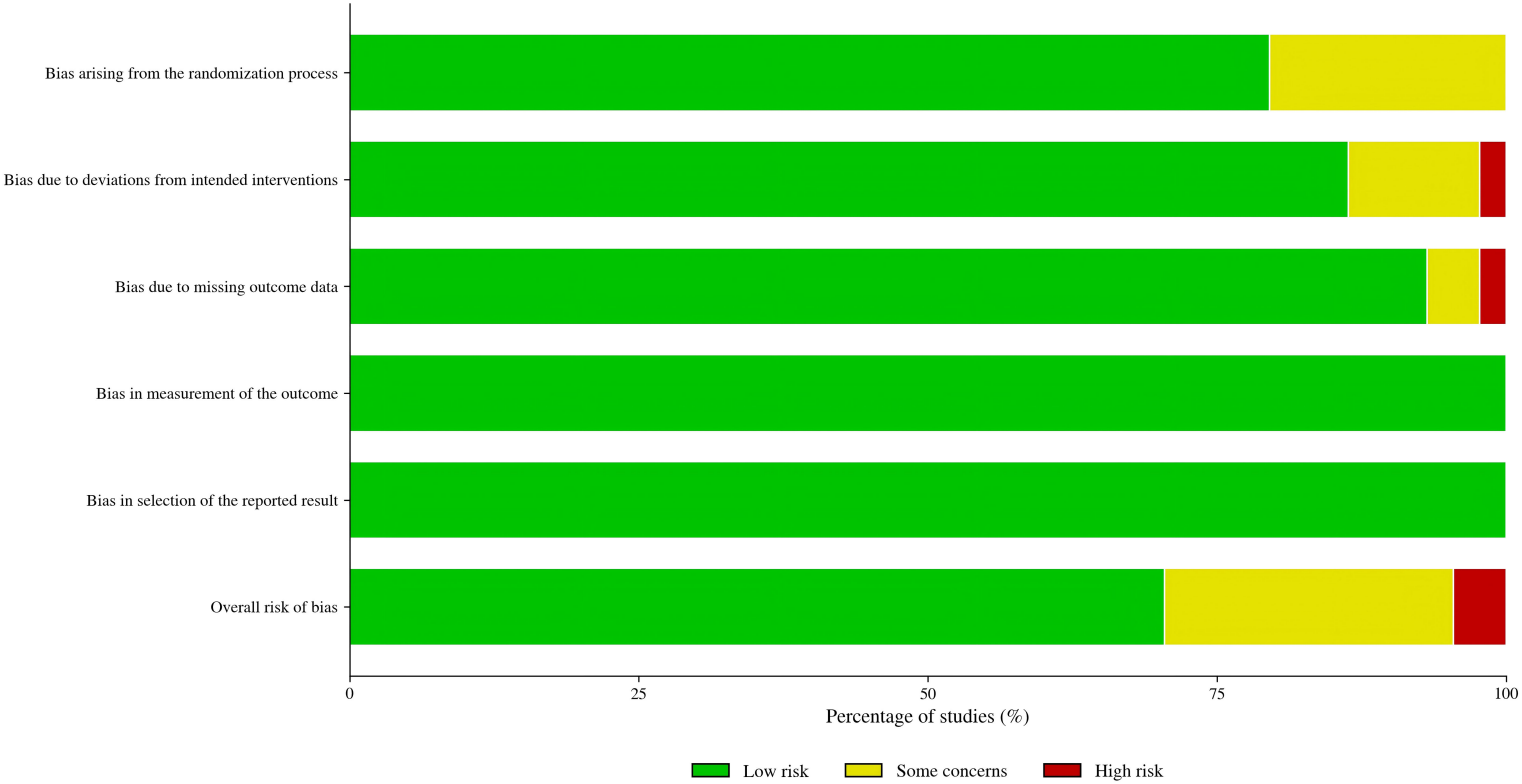
Distribution of studies across bias ranking.

The risk-of-bias assessment results for the included studies are shown in **Figures 3** and **4**. Most studies were rated as low risk in the domains of the randomization process and missing outcome data. Because complete blinding is difficult to implement in exercise interventions, and some outcomes relied on self-report scales, there were moderate-risk ratings in the domains of deviations from intended interventions and measurement of the outcome. The overall risk of bias did not indicate systematic high risk.

**Figure 5:**
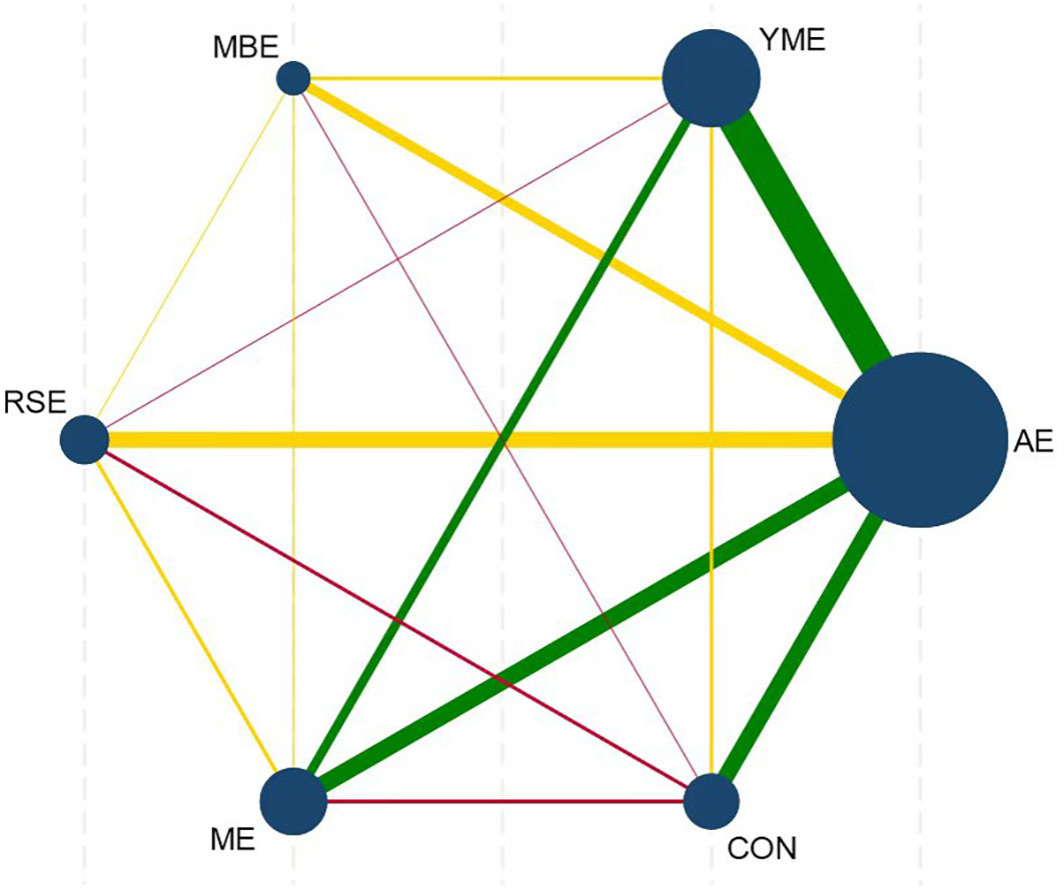
Risk-of-bias-based evidence network diagram of included studies.

Building on the overall risk-of-bias assessment, this study further constructed a risk-of-bias–based evidence network diagram to display the distribution of risk of bias across the different exercise intervention nodes and direct comparisons (**Figure 5**). The color composition within each node indicates the proportion of studies involving that exercise type rated as low risk of bias, some concerns, and high risk of bias; the color of each edge indicates the overall risk-of-bias level of the corresponding direct comparison, with green representing low risk of bias and yellow representing some concerns. No direct comparison was judged to be at high risk of bias. This risk-of-bias evidence network was used to support the interpretation of evidence credibility across intervention nodes and direct comparisons, with greater caution applied to comparisons involving fewer studies or a larger proportion of studies rated as having some concerns. Accordingly, confidence in the findings was considered stronger for comparisons supported by more direct evidence and consistent network estimates, whereas rankings based on sparse nodes, wider confidence intervals, or predominantly self-reported outcomes were interpreted more cautiously. Confidence in the network meta-analysis estimates was further appraised using the CINeMA framework; it was rated low for aerobic and multicomponent exercise versus control for addictive craving and for aerobic exercise versus control for negative affect, and very low for the remaining comparisons, mainly reflecting imprecision and the within-study limitations inherent to exercise trials (see **Table 2**).

**Table 2:** CINeMA confidence assessment for primary exercise-versus-control comparisons.

Outcome	Comparison	Number of studies	Within-study bias	Reporting bias	Indirectness	Imprecision	Heterogeneity	Incoherence	Confidence rating
O1 Addictive craving	AE vs CON	15	Some concerns	No concerns	No concerns	No concerns	Some concerns	No concerns	Low
	YME vs CON	2	Some concerns	Some concerns	No concerns	Major concerns	Some concerns	No concerns	Very low
	MBE vs CON	6	Some concerns	No concerns	No concerns	Major concerns	Some concerns	No concerns	Very low
	RSE vs CON	7	Some concerns	No concerns	No concerns	Major concerns	Some concerns	No concerns	Very low
	ME vs CON	8	Some concerns	No concerns	No concerns	No concerns	Some concerns	No concerns	Low
O2 Negative affect	AE vs CON	8	Some concerns	No concerns	No concerns	No concerns	Some concerns	No concerns	Low
	YME vs CON	5	Some concerns	No concerns	No concerns	Major concerns	Some concerns	No concerns	Very low
	MBE vs CON	8	Some concerns	No concerns	No concerns	Major concerns	Some concerns	No concerns	Very low
	RSE vs CON	5	Some concerns	No concerns	No concerns	Major concerns	Some concerns	No concerns	Very low
	ME vs CON	5	Some concerns	No concerns	No concerns	Some concerns	Some concerns	No concerns	Very low
O3 Cognitive function	AE vs CON	15	Some concerns	No concerns	Some concerns	Major concerns	Major concerns	No concerns	Very low
	YME vs CON	4	Some concerns	No concerns	Some concerns	Major concerns	Major concerns	No concerns	Very low
	MBE vs CON	3	Some concerns	Some concerns	Some concerns	Major concerns	Major concerns	No concerns	Very low
	RSE vs CON	8	Some concerns	No concerns	Some concerns	Major concerns	Major concerns	No concerns	Very low
	ME vs CON	5	Some concerns	No concerns	Some concerns	Major concerns	Major concerns	No concerns	Very low

Green shading indicates “no concerns,” yellow shading indicates “some concerns,” and red shading indicates “major concerns” in the CINeMA domain-level assessments. The unshaded final column presents the overall confidence rating.

### 3.4 Effects of different exercise interventions in patients with substance use disorder

#### 3.4.1 Results of the global inconsistency tests

To evaluate the reliability of the network meta-analysis, global inconsistency tests and the node-splitting method were applied separately to the three evidence networks of O1, O2, and O3 to assess internal consistency. The global inconsistency tests showed P values > 0.05 for all three networks (O1, O2, and O3), indicating no significant inconsistency and suggesting good agreement between the direct and indirect evidence in the three networks.

In addition, node-splitting analysis was performed for all nodes in each evidence network (see **Table 3**). The results showed that, in the O1 network, the differences between the direct and indirect effects for each splittable node did not reach statistical significance, with P values ranging from 0.111 to 0.989; in the O2 network, the direct–indirect differences for each comparison likewise did not reach statistical significance, with P values ranging from 0.095 to 0.945; and in the O3 network, the node-splitting test results also showed no significant local inconsistency, with P values ranging from 0.487 to 0.990. These results indicate that no significant systematic discrepancy was found between the direct and indirect evidence in the three networks, supporting the use of a consistency model for the subsequent network meta-analysis.

**Table 3:** Node-splitting tests of direct–indirect consistency across the three outcome networks.

Outcome	Treatment comparison	Direct estimate (SE)	Indirect estimate (SE)	Difference, direct-indirect (SE)	P value for inconsistency
Outcome 1					
	AE vs RSE	0.242 (0.489)	0.252 (0.557)	−0.010 (0.742)	0.989
	AE vs CON	0.759 (0.254)	1.093 (1.301)	−0.334 (1.326)	0.801
	YME vs MBE	−0.011 (0.972)	−0.993 (0.885)	0.983 (1.315)	0.455
	YME vs RSE	−1.027 (0.961)	−0.353 (0.871)	−0.674 (1.297)	0.603
	YME vs CON	−0.209 (0.691)	0.147 (1.319)	−0.356 (1.490)	0.811
	MBE vs RSE	−0.257 (0.956)	−0.061 (0.544)	−0.196 (1.100)	0.859
	MBE vs ME	−0.070 (0.952)	−0.745 (0.552)	0.675 (1.101)	0.540
	MBE vs CON	0.346 (0.388)	1.148 (1.264)	−0.802 (1.324)	0.545
	RSE vs ME	0.091 (0.665)	−0.848 (0.555)	0.940 (0.866)	0.278
	RSE vs CON	0.277 (0.350)	1.559 (0.722)	−1.281 (0.804)	0.111
	ME vs CON	0.983 (0.346)	1.108 (1.210)	−0.125 (1.258)	0.921
Outcome 2					
	AE vs YME	−0.336 (1.066)	0.633 (0.669)	−0.969 (1.259)	0.442
	AE vs MBE	0.043 (1.078)	1.012 (0.557)	−0.970 (1.214)	0.424
	AE vs RSE	1.833 (0.770)	0.524 (0.708)	1.310 (1.049)	0.212
	AE vs ME	0.480 (0.778)	0.201 (0.752)	0.279 (1.083)	0.796
	AE vs CON	1.191 (0.394)	1.506 (1.015)	−0.314 (1.088)	0.773
	YME vs CON	0.731 (0.476)	3.265 (1.967)	−2.534 (2.023)	0.211
	MBE vs RSE	0.387 (1.094)	0.299 (0.647)	0.087 (1.271)	0.945
	MBE vs ME	0.320 (1.073)	−0.770 (0.657)	1.090 (1.259)	0.386
	MBE vs CON	0.254 (0.365)	2.465 (1.274)	−2.211 (1.325)	0.095
	RSE vs CON	0.329 (0.473)	−1.463 (1.250)	1.792 (1.338)	0.181
	ME vs CON	0.931 (0.494)	0.637 (1.370)	0.294 (1.461)	0.840
Outcome 3					
	AE vs YME	1.400 (3.015)	1.680 (4.184)	−0.279 (5.158)	0.957
	AE vs RSE	0.245 (2.330)	1.642 (3.017)	−1.397 (3.812)	0.714
	AE vs ME	1.081 (3.680)	2.967 (2.889)	−1.886 (4.678)	0.687
	AE vs CON	2.540 (1.365)	2.197 (5.299)	0.343 (5.472)	0.950
	YME vs ME	3.705 (5.163)	−0.650 (3.560)	4.355 (6.271)	0.487
	YME vs CON	0.493 (2.598)	3.396 (5.502)	−2.903 (6.083)	0.633
	MBE vs RSE	0.327 (3.688)	−0.873 (4.724)	1.199 (5.993)	0.841
	MBE vs ME	1.843 (3.690)	0.334 (5.329)	1.509 (6.482)	0.816
	MBE vs CON	1.025 (3.001)	4.169 (6.191)	−3.144 (6.880)	0.648
	RSE vs ME	1.516 (3.693)	1.456 (3.245)	0.060 (4.916)	0.990
	RSE vs CON	1.262 (1.834)	4.500 (4.357)	−3.239 (4.727)	0.493
	ME vs CON	0.011 (2.335)	1.346 (4.771)	−1.335 (5.312)	0.802

#### 3.4.2 Network meta-analysis results under the consistency model

Based on the inconsistency test results, a consistency model was used to conduct the network meta-analysis of the three evidence networks. According to the edge structure for which direct evidence was available in the node-splitting table, the O1 network formed six independent closed loops (AE–CON–RSE–AE, RSE–CON–MBE–RSE, RSE–CON–ME–RSE, RSE–CON–YME–RSE, MBE–CON–YME–MBE, and MBE–CON–ME–MBE), the O2 network formed six independent closed loops (AE–CON–YME–AE, AE–CON–RSE–AE, AE–CON–ME–AE, AE–CON–MBE–AE, RSE–CON–MBE–RSE, and ME–CON–MBE–ME), and the O3 network formed seven independent closed loops (AE–CON–YME–AE, AE–CON–RSE–AE, AE–CON–ME–AE, YME–CON–ME–YME, MBE–CON–RSE–MBE, MBE–CON–ME–MBE, and RSE–CON–ME–RSE), indicating that all three networks met the basic conditions for integrating direct and indirect evidence. With respect to local inconsistency, none of the comparisons amenable to node-splitting tests in the O1, O2, and O3 networks showed a significant difference between direct and indirect evidence, suggesting that no significant local inconsistency was found in any of the three networks.

#### 3.4.3 SUCRA ranking results of the network meta-analysis

**Figure 6:**
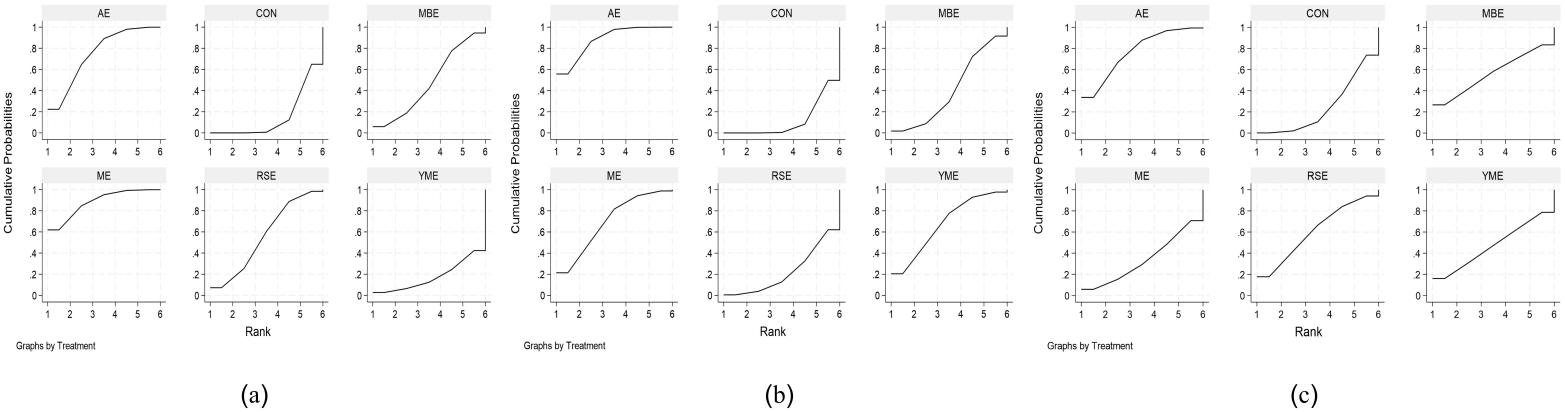
SUCRA cumulative ranking curves. **(a)** addictive craving (O1), **(b)** negative affect (O2), and **(c)** cognitive function (O3).

This study further calculated the SUCRA values of each exercise intervention in the O1, O2, and O3 networks to quantify the relative ranking of the five types of exercise interventions and the control condition (see **Figure 6**, **Table 4**). A higher SUCRA value indicates a more favorable ranking of the intervention in the corresponding outcome; the specific effect size needs to be interpreted in conjunction with the SMD and its 95% CI.

**Table 4:** Surface under the cumulative ranking curve (SUCRA) of five exercise modalities across craving, affect, and cognitive control networks.

Intervention	Outcome 1		Outcome 2		Outcome 3	
	Rank	SUCRA	Rank	SUCRA	Rank	SUCRA
AE	2	0.749	1	0.880	1	0.770
YME	5	0.177	3	0.675	4	0.473
MBE	4	0.477	4	0.408	3	0.562
RSE	3	0.561	5	0.223	2	0.609
ME	1	0.882	2	0.697	5	0.340
CON	6	0.156	6	0.117	6	0.247

In the O1 network, ME ranked highest (SUCRA = 0.882), followed by AE (SUCRA = 0.749) and RSE (SUCRA = 0.561), with MBE in the middle (SUCRA = 0.477), and YME (SUCRA = 0.177) and CON (SUCRA = 0.156) ranking lowest. This ranking was broadly consistent with the direction of the pooled effects relative to CON: both ME and AE showed a statistically significant advantage relative to CON, the effect direction of RSE and MBE relative to CON was positive but did not reach statistical significance, and YME did not reach statistical significance relative to CON.

In the O2 network, AE ranked first (SUCRA = 0.880), with ME (SUCRA = 0.697) and YME (SUCRA = 0.675) ranking second and third; MBE (SUCRA = 0.408) and RSE (SUCRA = 0.223) ranked relatively low, and CON (SUCRA = 0.117) was last. In conjunction with the pairwise comparison results, AE and ME reached statistical significance relative to CON; although YME ranked relatively high, its 95% CI crossed zero and should be interpreted with caution. No clear statistical difference was observed among the top three interventions in O2, suggesting that AE, ME, and YME all showed relatively high rankings for improving negative affective symptoms, with AE ranking first.

In the O3 network, AE ranked highest (SUCRA = 0.770), with RSE (SUCRA = 0.609) and MBE (SUCRA = 0.562) ranking second and third, YME (SUCRA = 0.473) in the middle, and ME (SUCRA = 0.340) and CON (SUCRA = 0.247) ranking relatively low.

In summary, the relative rankings of the different exercise interventions differed somewhat across the three outcomes. For the O1 outcome, ME performed relatively well, and AE also showed a high ranking and a significant effect; for the O2 outcome, AE, ME, and YME ranked high, with the effects of AE and ME relative to CON being more clearly defined; and for the cognitive function outcome, AE showed a relatively stable ranking advantage, but, because of the wide confidence intervals, the relevant conclusions still require further verification.

#### 3.4.4 Pairwise meta-analytic comparisons

The pairwise comparison results among the interventions under the consistency model are shown in **Table 5**. All results are expressed as the SMD with its 95% CI, where an SMD > 0 indicates that the column-defining intervention has a more favorable improvement effect than the row-defining intervention.

**Table 5:** League tables of relative treatment effects for craving, negative affect, and cognitive control outcomes.

Panel A. Craving symptoms, O1						
Row-defining intervention	ME	AE	RSE	MBE	YME	CON
ME	—					
AE	0.220 (−0.570, 1.000)	—				
RSE	0.460 (−0.370, 1.300)	0.250 (−0.460, 0.950)	—			
MBE	0.570 (−0.350, 1.490)	0.350 (−0.500, 1.210)	0.110 (−0.800, 1.020)	—		
YME	1.120 (−0.200, 2.440)	0.900 (−0.350, 2.160)	0.660 (−0.590, 1.900)	0.550 (−0.720, 1.820)	—	
CON	0.990 (0.350, 1.630)	0.770 (0.290, 1.250)	0.520 (−0.110, 1.160)	0.420 (−0.300, 1.130)	−0.130 (−1.310, 1.040)	—
Panel B. Negative affect symptoms, O2						
Row-defining intervention	AE	ME	YME	MBE	RSE	CON
AE	—					
ME	0.330 (−0.700, 1.370)	—				
YME	0.360 (−0.740, 1.460)	0.020 (−1.240, 1.290)	—			
MBE	0.810 (−0.150, 1.770)	0.470 (−0.620, 1.560)	0.450 (−0.710, 1.600)	—		
RSE	1.130 (0.110, 2.150)	0.790 (−0.430, 2.010)	0.770 (−0.480, 2.020)	0.320 (−0.750, 1.390)	—	
CON	1.230 (0.520, 1.940)	0.890 (0.010, 1.780)	0.870 (−0.040, 1.790)	0.420 (−0.290, 1.140)	0.100 (−0.770, 0.970)	—
Panel C. Cognitive control function, O3						
Row-defining intervention	AE	RSE	MBE	YME	ME	CON
AE	—					
RSE	0.760 (−2.780, 4.300)	—				
MBE	0.890 (−4.720, 6.490)	0.130 (−5.450, 5.700)	—			
YME	1.490 (−3.200, 6.180)	0.730 (−4.660, 6.110)	0.600 (−6.170, 7.370)	—		
ME	2.240 (−2.120, 6.610)	1.480 (−3.190, 6.150)	1.360 (−4.460, 7.170)	0.750 (−4.920, 6.430)	—	
CON	2.510 (−0.020, 5.040)	1.750 (−1.520, 5.010)	1.620 (−3.570, 6.820)	1.020 (−3.500, 5.540)	0.270 (−3.760, 4.290)	—

In the addictive craving symptom (O1) network, ME and AE showed a statistically significant advantage relative to CON, with ME (SMD = 0.990, 95% CI: 0.350, 1.630) having a higher effect size, followed by AE (SMD = 0.770, 95% CI: 0.290, 1.250); RSE (SMD = 0.520, 95% CI: −0.110, 1.160), MBE (SMD = 0.420, 95% CI: −0.300, 1.130), and YME (SMD = −0.130, 95% CI: −1.310, 1.040) did not reach statistical significance relative to CON. Comparisons among the exercise interventions showed no clear statistical differences between any of the exercise types.

In the negative affective symptom (O2) network, AE (SMD = 1.230, 95% CI: 0.520, 1.940) and ME (SMD = 0.890, 95% CI: 0.010, 1.780) reached statistical significance relative to CON; YME (SMD = 0.870, 95% CI: −0.040, 1.790), MBE (SMD = 0.420, 95% CI: −0.290, 1.140), and RSE (SMD = 0.100, 95% CI: −0.770, 0.970) did not reach significance relative to CON. Comparisons among the exercise interventions showed that AE was significantly superior to RSE (SMD = 1.130, 95% CI: 0.110, 2.150), with no significant differences observed among the other exercise interventions, suggesting that AE and ME have relatively more clearly defined benefits for improving negative affective symptoms.

In the cognitive function (O3) network, AE showed a relatively large positive effect trend relative to CON (SMD = 2.510, 95% CI: −0.020, 5.040); RSE (SMD = 1.750, 95% CI: −1.520, 5.010), MBE (SMD = 1.620, 95% CI: −3.570, 6.820), YME (SMD = 1.020, 95% CI: −3.500, 5.540), and ME (SMD = 0.270, 95% CI: −3.760, 4.290) likewise did not reach statistical significance relative to CON. These pairwise results indicate that the statistically supported effects should be interpreted according to the specified comparator, particularly comparisons with CON, and in conjunction with SUCRA rankings.

#### 3.4.5 Sensitivity analysis

To test the robustness of the main analysis, sensitivity analyses were conducted separately for the three outcome networks, with a focus on comparing whether the effect direction, the pattern of statistical significance, and the SUCRA ranking framework of the main interventions relative to CON changed substantively before and after the sensitivity analyses. The results showed that the main conclusions of the three outcome networks remained largely stable. Specifically, in the addictive craving symptom (O1) network, ME and AE still maintained relatively clear positive effect signals relative to CON, and the ranking pattern of the main advantageous interventions did not change in direction; in the negative affective symptom (O2) network, AE and ME remained the relatively better-performing intervention types, with the overall effect direction consistent with the main analysis; and in the cognitive function (O3) network, none of the exercise interventions showed a stable statistically significant advantage relative to CON, but AE still maintained a high ranking and a positive effect trend. Overall, the sensitivity analyses did not change the judgments of this study regarding the main advantageous interventions and their outcome-specific performance across the three outcomes, suggesting that the main analysis results are reasonably robust.

### 3.5 Neuropharmacological substance-subgroup stratified analysis of the craving outcome

**Figure 7:**
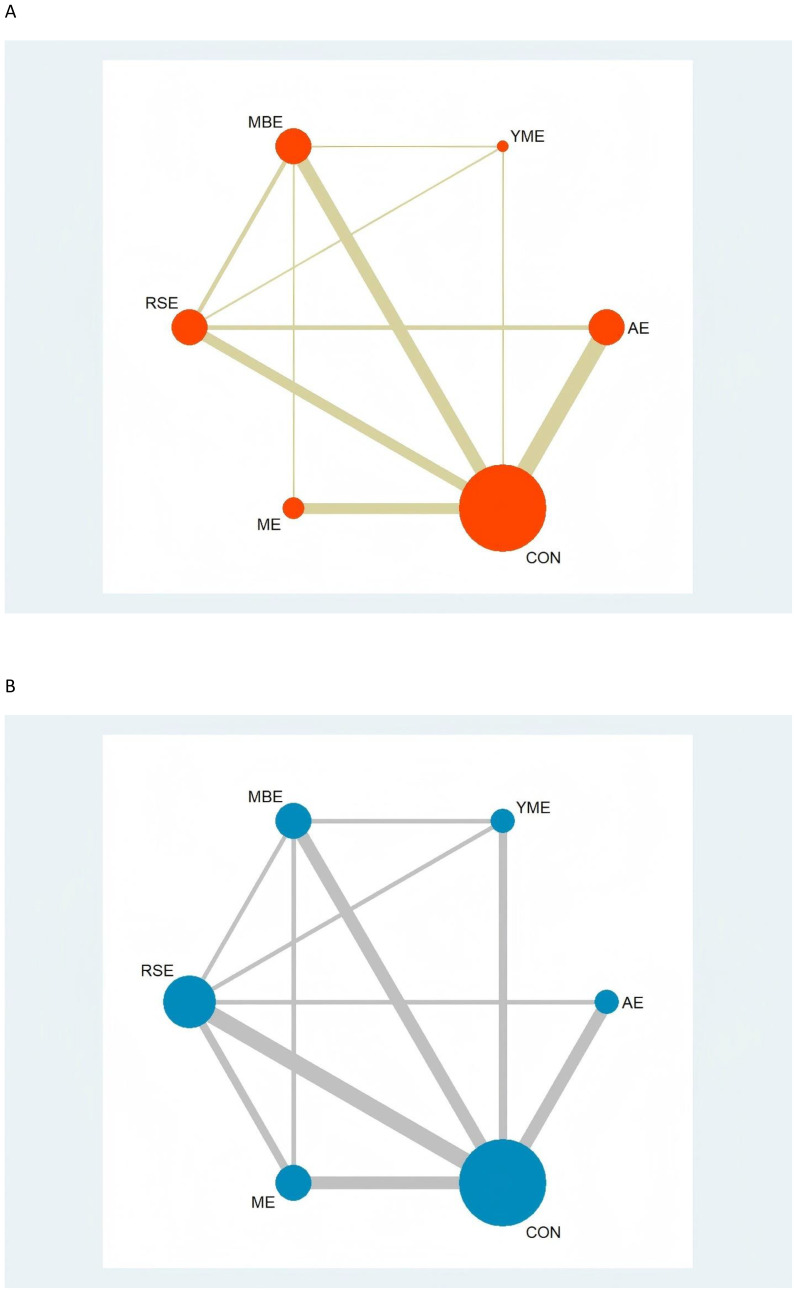
**(A)** StUD subgroup network plot for O1. **(B)** Non-StUD subgroup network plot for O1.

Given that addictive craving is closely related to reward sensitization, motivational drive, withdrawal-induced urges, and relapse risk, this study further conducted a neuropharmacological substance-subgroup stratified analysis within the O1 network. According to the main substance used as reported in the original studies, the studies included in the O1 network were divided into two subgroups, stimulant use disorder (StUD) and non-stimulant use disorder (Non-StUD), and independent NMA models were fitted for each (see **Figures 7A, B**).

#### 3.5.1 Stimulant use disorder subgroup

A global inconsistency test for the StUD subgroup indicated no significant inconsistency (P > 0.05); therefore, a consistency model was used for the analysis.

The results showed that, relative to CON, ME, AE, and MBE all showed a statistically significant advantage, with effect sizes of ME (SMD = 1.330, 95% CI: 0.090, 2.580), AE (SMD = 1.030, 95% CI: 0.030, 2.020), and MBE (SMD = 1.120, 95% CI: 0.010, 2.240). RSE (SMD = 0.540, 95% CI: −0.650, 1.730) and YME (SMD = 0.540, 95% CI: −1.860, 2.940) did not reach statistical significance relative to CON.

At the SUCRA ranking level, ME (SUCRA = 0.748) ranked highest in the StUD subgroup, with MBE (SUCRA = 0.675) and AE (SUCRA = 0.638) ranking second and third, YME (SUCRA = 0.430) and RSE (SUCRA = 0.395) ranking lower, and CON (SUCRA = 0.117) last. This suggests that, among people with stimulant addiction, interventions such as ME and MBE—which integrate multichannel stimuli or emphasize mind–body regulation—may be more likely to improve addictive craving symptoms than purely strength-based stimulation (RSE) or purely attention–awareness training (YME).

#### 3.5.2 Non-stimulant use disorder subgroup

A global inconsistency test for the Non-StUD subgroup indicated no significant inconsistency (P > 0.05); a consistency model was used for the analysis.

The results showed that, relative to CON, only AE showed a statistically significant advantage (SMD = 0.53, 95% CI: 0.03, 1.03). RSE (SMD = 0.39, 95% CI: −0.05, 0.82), ME (SMD = 0.31, 95% CI: −0.21, 0.82), MBE (SMD = 0.23, 95% CI: −0.28, 0.74), and YME (SMD = −0.23, 95% CI: −0.88, 0.41) did not reach statistical significance relative to CON.

The SUCRA rankings showed that AE (SUCRA = 0.837) ranked first, RSE (SUCRA = 0.707) ranked second, ME (SUCRA = 0.604) and MBE (SUCRA = 0.523) ranked third and fourth, and CON (SUCRA = 0.224) and YME (SUCRA = 0.105) ranked lower. This suggests that AE and RSE have relatively favorable effects on improving addictive craving in patients with non-stimulant use disorder.

#### 3.5.3 Comparison of SUCRA rankings between the StUD and non-StUD subgroups

**Figure 8:**
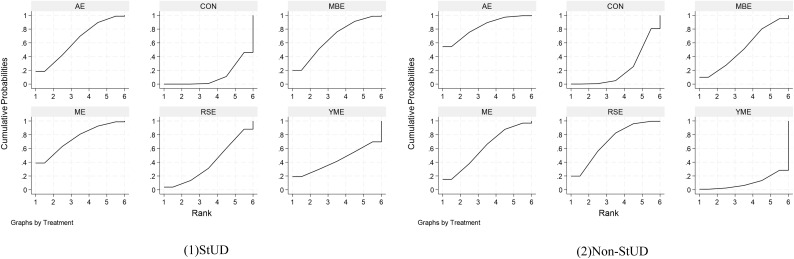
SUCRA cumulative ranking curves for exercise interventions on craving symptoms in StUD and Non-StUD subgroups.

The cumulative SUCRA ranking probabilities and ranking results for each intervention in the StUD and Non-StUD subgroups are shown in **Figure 8** and **Table 6**. Overall, the intervention rankings of the two subgroups were not entirely consistent. In the StUD subgroup, the cumulative ranking curves of ME and MBE were located in relatively favorable positions overall, with SUCRA values of 0.748 and 0.675, respectively, suggesting that both ranked relatively high for improving addictive craving in patients with stimulant use disorder. AE ranked third (SUCRA = 0.638), while YME (SUCRA = 0.430) and RSE (SUCRA = 0.395), although ranking relatively lower, were still better than CON (SUCRA = 0.117).

**Table 6:** SUCRA rankings of exercise interventions on craving (O1) within StUD and Non-StUD subgroups.

Intervention	StUD subgroup SUCRA	Non-StUD subgroup SUCRA
ME	0.748 (rank 1)	0.604 (rank 3)
MBE	0.675 (rank 2)	0.523 (rank 4)
AE	0.638 (rank 3)	0.837 (rank 1)
RSE	0.395 (rank 5)	0.707 (rank 2)
YME	0.430 (rank 4)	0.105 (rank 6)
CON	0.117 (rank 6)	0.224 (rank 5)

In the Non-StUD subgroup, the ranking distribution of the exercise interventions showed a different pattern from that of the StUD subgroup. AE had the highest SUCRA value (SUCRA = 0.837), RSE ranked second (SUCRA = 0.707), ME ranked third (SUCRA = 0.604), and MBE ranked fourth (SUCRA = 0.523); CON (SUCRA = 0.224) and YME (SUCRA = 0.105) ranked lower. These results suggest that, among patients with non-stimulant use disorder, AE and RSE ranked relatively higher for improving addictive craving.

From the pairwise subgroup comparison, AE ranked third in the StUD subgroup but rose to first in the Non-StUD subgroup, suggesting that aerobic exercise may have a relatively stable and prominent potential to improve addictive craving across patients with different substance subtypes. By contrast, ME and MBE ranked higher in the StUD subgroup, in first and second place, respectively, whereas RSE rose to second place in the Non-StUD subgroup, indicating that the relative advantages of different exercise types may differ across patients with different substance subtypes. Overall, the StUD subgroup tended to favor higher rankings for ME and MBE, whereas the Non-StUD subgroup favored higher rankings for AE and RSE.

#### 3.5.4 Robustness verification of the substance-subgroup analysis

To further verify the stability of the substance-subgroup classification, this study conducted a robustness analysis of the Non-StUD subgroup. Specifically, building on the main analysis, the Non-StUD subgroup network model was refitted after excluding five studies that described participants only with general terms such as “drug,” “drug use,” or similar wording. The results showed that, after excluding these studies, the effect directions of the main interventions relative to CON remained consistent with the main analysis, RSE and AE still showed relatively stable directions of advantage, and the main ranking pattern did not change. These results indicate that the Non-StUD subgroup findings were not noticeably affected by differences in the description of substance type, supporting the appropriateness of conducting the StUD and Non-StUD subgroup analyses based on neuropharmacological classification in this study.

## 4 Discussion

This study systematically compared the relative effects of five types of exercise interventions on three outcomes—addictive craving, negative affect, and cognitive function—and further conducted a neuropharmacological substance-subtype stratified analysis for the craving outcome. The results showed that the relative performance of the different exercise interventions differed across the three symptom outcomes, and that the pattern of exercise response in the craving outcome may be influenced by the main substance type used. Overall, the findings suggest that the effects of exercise-based rehabilitation in SUD need to be formulated by integrating symptom target, substance type, and exercise mechanism; this is discussed below in terms of differentiated prescriptions across the three outcomes, substance-subgroup differences within the craving outcome, and the refinement of precision exercise prescriptions.

### 4.1 Differences in the performance of the same exercise type across outcomes

The results showed that the relative rankings of the same exercise type were not consistent across the three outcomes. Aerobic exercise (AE) ranked first in both O2 and O3 and second in O1. Multicomponent exercise (ME) ranked first in O1 and second in O2 but relatively low in O3. These across-outcome ranking fluctuations reflect varying degrees of alignment between the action pathways of each exercise modality and the neural substrates underlying each symptom dimension. These outcome-specific patterns may also be biologically plausible, because exercise can act on partially overlapping pathways related to reward processing, stress regulation, mood improvement, prefrontal cognitive regulation, and craving relief, with different exercise modalities emphasizing these pathways to varying degrees.

The neural basis of addictive craving lies in the coupled imbalance of reward-circuit sensitization (the VTA–NAc dopaminergic projection), enhanced cue reactivity (the amygdala–insula), and a decline in prefrontal inhibitory control (7, 17). This implies that an exercise with relatively good effects at the level of craving needs to possess the dual actions of “reward-system rebalancing” and “prefrontal control reconstruction.” By integrating aerobic, resistance, and coordination components, ME may simultaneously mobilize monoaminergic neurotransmitter release, BDNF-mediated neuroplasticity, and the prefrontal–cerebellar circuit (71, 73); AE, in turn, may promote reward-system rebalancing and overall improvement in physical and mental status through improved cardiorespiratory fitness, monoaminergic neurotransmitter modulation, and activation of the endogenous opioid system (72, 73). The two have different pathways of action but can both achieve improvement in addictive craving symptoms. Although the effect direction of MBE was positive in the overall O1 network, it did not reach statistical significance relative to CON, and its effect may be more apparent in specific substance subgroups. By contrast, the YME interventions in this study were mostly based on yoga postures and simplified mindfulness, and their neural effects lean more toward the attention–emotion regulation pathway (69, 74), with relatively limited direct evidence for intervention on reward-system sensitization, which may be the underlying reason for its poor performance in O1.

The neural basis of negative affect, in turn, centers on overactivation of the HPA axis, depletion of monoaminergic neurotransmitters, and a decline in BDNF (4, 72). This pathology is relatively sensitive to both the bodily activation pathway (the metabolic–monoaminergic cascade of AE (72, 73)) and the emotion-regulation pathway (the remodeling of prefrontal–amygdala functional connectivity by YME), but in this study only AE and ME reached statistical significance relative to CON. The exercise intensity of MBE is usually insufficient to fully activate the metabolic pathway, and the direct regulatory effect of RSE on the monoaminergic system and the HPA axis is relatively weak (11); both therefore showed relatively limited performance on this outcome.

The neural basis of cognitive control decline is functional impairment of the prefrontal–dorsal striatal–ACC circuit and reduced neurogenesis in the hippocampal dentate gyrus (7); its improvement depends heavily on cardiorespiratory-fitness-driven upregulation of BDNF/VEGF and increased prefrontal–hippocampal cerebral blood perfusion (72, 73), which is precisely the mechanistic basis for AE ranking highest and showing a relatively stable effect signal in O3. In addition, the decline in ME performance in O3 may reflect a dose-dilution phenomenon: within a limited intervention time, multichannel training disperses the dose concentration required for high-intensity aerobic or dedicated cognitive–motor coupling training. This suggests that multicomponent integration is not superior to single-component training for all outcomes; the key lies in whether the core pathological pathway of the given outcome requires concentrated intervention.

### 4.2 Substance-subgroup-related differences in response within the craving outcome

This study further found that, in the craving outcome, the intervention rankings and significance patterns of the StUD and Non-StUD subgroups were not entirely consistent. In the StUD subgroup, MBE, ME, and AE reached statistical significance relative to CON, with ME and MBE ranking high; in the Non-StUD subgroup, AE reached statistical significance relative to CON, whereas RSE, MBE, and ME had positive point estimates but did not reach statistical significance, indicating that the relative effects of exercise interventions on addictive craving may be influenced by the main substance type used.

This difference can be explained to some extent by the patterns of neuropharmacological impairment of different substance categories. Stimulant use disorders, especially methamphetamine- and cocaine-related disorders, are typically associated with abnormalities in monoaminergic systems such as dopamine and norepinephrine, sensitization of reward pathways, enhanced cue-induced reactivity, and impaired prefrontal inhibitory control. The interventions required for this pathology are precisely those that “reduce overarousal and reconstruct prefrontal top-down control”: MBE reduces sympathetic tone through vagal activation and strengthens interoceptive–prefrontal connectivity via the insula (70), while ME simultaneously mobilizes dopaminergic rebalancing and coordination–cognition coupling through multichannel stimulation; although their mechanistic pathways differ, both are consistent with the dual pathological features of StUD. In this context, exercise forms that can simultaneously influence body awareness, emotional reactivity, autonomic activity, and behavioral control may be more conducive to regulating craving-related overreactivity. The movement–breath–awareness integration features of MBE may help reduce overarousal and enhance bodily interoceptive regulation and emotional stability; the composite training structure of ME may, through multichannel stimulation, influence neuroplasticity, emotion regulation, and the recovery of bodily function. Therefore, ME and MBE showed relatively good intervention effects in the StUD subgroup.

In the Non-StUD subgroup, AE showed a more clearly defined statistical advantage relative to CON, whereas RSE, although showing a positive point estimate, did not reach statistical significance relative to CON. Unlike the overactivation-type pathology of the StUD subgroup, which centers on overactivation of the central dopamine system and sensitization of reward pathways, the non-stimulant substances covered by Non-StUD differ in their specific receptor sites of action but share, at the clinical level, common “low-functioning-type” features such as low arousal, low motivation, anhedonia, and declining bodily function, suggesting that their reward–motivation systems are overall in a state of functional downregulation. This shared clinical feature may provide a common target for exercise intervention. That is, craving in patients with Non-StUD does not arise from systemic overactivation but rather from a “low-functioning state” of the motivational system, suggesting that the core mechanism of effective intervention should be behavioral activation and the reconstruction of self-efficacy, rather than mind–body awareness or attention training. RSE, through improvements in muscle strength, a sense of bodily control, and self-efficacy, may have strong behavioral-activation significance for patients with low vitality, low motivation, or declining bodily function (11, 12); AE, in turn, may broadly reconstruct physical and mental activation levels by activating the endogenous opioid system and improving cardiorespiratory fitness, thereby playing a broader role in improving cardiorespiratory function, promoting emotion regulation, and enhancing overall bodily status (71, 72). In this study, AE ranked first and RSE ranked second in the Non-StUD subgroup, suggesting that both may be more advantageous exercise interventions in this subgroup.

### 4.3 Interpretation of the intervention effects of different exercise types

Different exercise intervention programs may show differentiated effects in SUD recovery because of differences in training content, mode of participation, and patients’ symptom burden. Aerobic exercise (AE), characterized mainly by continuous cardiorespiratory activity, has strong operability and a broad scope of application. In this study, AE maintained relatively high rankings in O1, O2, and O3, ranking first in O2 and O3; in the substance-subgroup analysis, AE ranked third in the StUD subgroup and first in the Non-StUD subgroup, suggesting that it may be more suitable as a foundational exercise choice in SUD exercise-based rehabilitation, especially for patients with an unclear substance type, polysubstance use, or relatively complex symptom presentation.

Multicomponent exercise (ME), composed of multiple training components and usually including cardiorespiratory activity, strength training, coordination training, or other comprehensive content simultaneously, may therefore be more suitable for patients in whom craving, affective, and bodily function problems are intertwined. In this study, ME performed relatively well in both the addictive craving and negative affect outcomes, ranking first in O1 and second in O2, and showed a statistically significant advantage relative to CON in both, suggesting that comprehensive exercise programs may be more suitable for SUD rehabilitation scenarios with multiple coexisting symptoms.

Mind–body exercise (MBE), characterized mainly by movement control, coordinated breathing, and body awareness, shows its advantages more in regulating autonomic balance, reducing overarousal, and enhancing bodily interoception. In this study, the effect direction of MBE was positive in the overall O1 network but did not reach statistical significance relative to CON; in the StUD subgroup, however, it ranked high and reached statistical significance, suggesting that it may be more suitable for situations in patients with stimulant addiction in whom high arousal, strong cue reactivity, and difficulties with self-regulation are prominent.

Resistance exercise (RSE), characterized mainly by strength training and an enhanced sense of bodily control, did not show a clear advantage in the overall O1 and O2 networks in this study, but ranked second and reached statistical significance in the Non-StUD subgroup. This suggests that the effect of RSE may not be mainly reflected in broad-spectrum emotion regulation or reward rebalancing, but rather more likely produces behavioral-activation significance for patients with low vitality, low motivation, or declining bodily function through improvements in muscle strength, a sense of bodily control, and self-efficacy.

Yoga- or mindfulness-based exercise (YME), with yoga postures, breathing training, and mindfulness practice as its main content, leans more toward emotion regulation and stress relief. In this study, YME ranked third in O2, suggesting that it may have a certain potential for regulating negative affect, but its rankings in O1 and the Non-StUD subgroup were relatively low. The results indicate that YME showed a positive effect in improving negative affective symptoms, but its evidence signals in the craving and cognitive function outcomes were not stable, suggesting that its effects may be more concentrated at the affective level rather than having an equivalent effect on all core SUD symptoms.

Therefore, the differentiated performance of the different exercise types may reflect the degree of matching between the training content, behavioral demands, and the main symptom burden of each exercise modality. AE leans more toward foundational support, ME is more suitable for comprehensive intervention where craving and negative affect are intertwined, MBE may be more applicable for craving improvement in patients with StUD, RSE may be more suitable for behavioral activation and the reconstruction of self-efficacy in patients with Non-StUD, and YME can serve as a complementary option for negative affect regulation.

### 4.4 Implications for precision-oriented exercise-based rehabilitation

**Figure 9:**
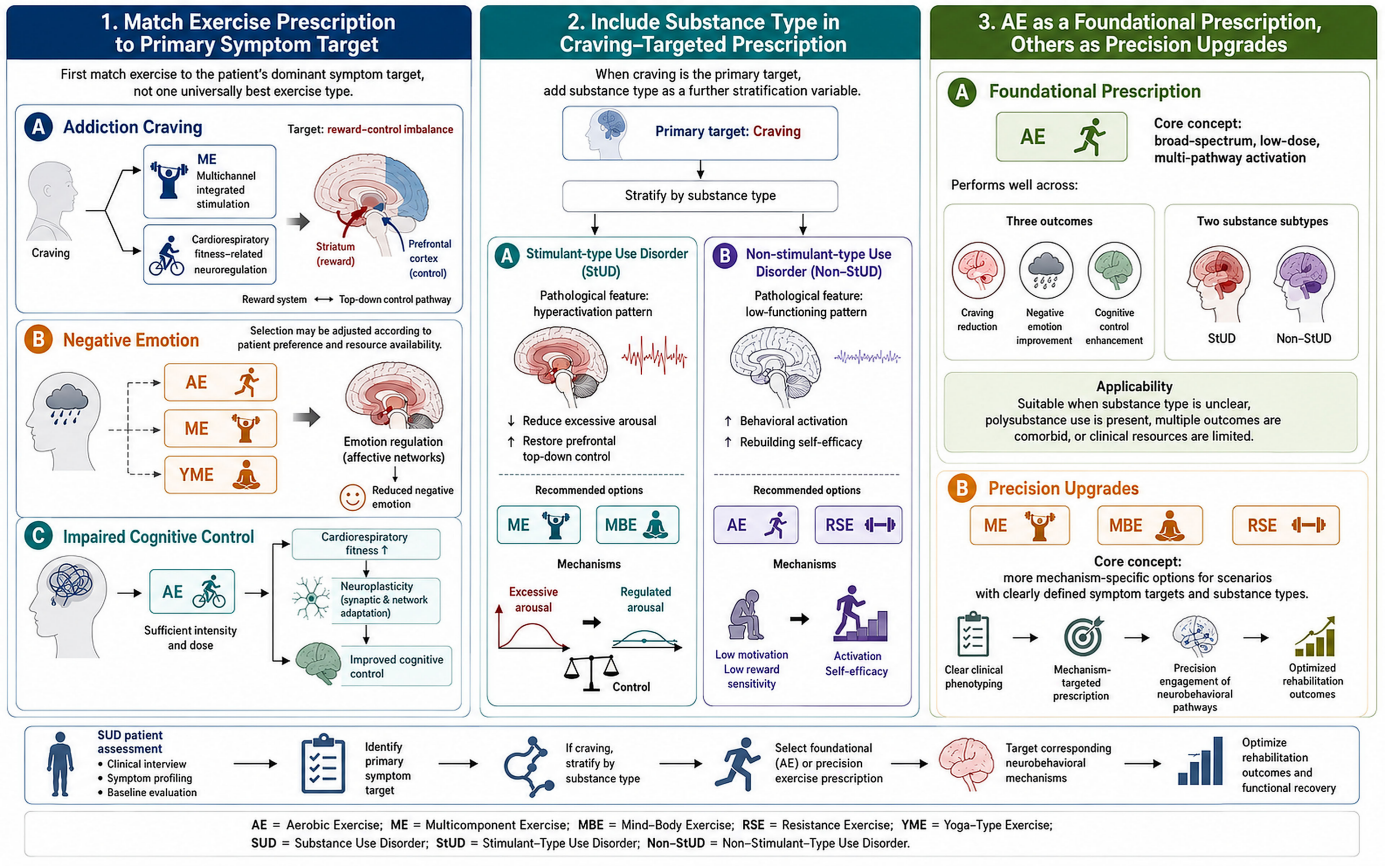
Symptom target–substance type–exercise mechanism matching framework for exercise prescriptions in substance use disorder.

The findings of this study have three implications for the design of SUD exercise-based rehabilitation programs (**Figure 9**). First, exercise prescriptions should preferentially match the exercise program to the patient’s main symptom target, rather than focusing exclusively on identifying a single optimal exercise type. For patients whose main intervention target is addictive craving, ME and AE have more clearly defined improvement effects in the overall network, hitting the reward–control imbalance pathology of craving through multichannel integrated stimulation and cardiorespiratory-fitness-related neural modulation, respectively; if substance subgroups are further distinguished, ME and MBE may be emphasized for patients with StUD, and AE and RSE for patients with Non-StUD. For patients whose main intervention target is negative affect, AE and ME may serve as preferred options and YME as a complementary option, with the clinical choice determined in conjunction with patient preference and resource conditions. For patients whose main intervention target is declining cognitive function, aerobic exercise at a training intensity and dose sufficient to trigger the “cardiorespiratory fitness–neuroplasticity” pathway may be adopted.

Second, when craving is the intervention target, substance type should be incorporated into prescription decisions as a further stratification variable. Stimulant addiction presents an “overactivation-type” pathology and may be addressed with ME and MBE to “reduce overarousal and reconstruct prefrontal top-down control.” Non-stimulant addiction, in turn, presents a “low-functioning-type” pathology and may be addressed with AE and RSE in an intervention centered on behavioral activation and the reconstruction of self-efficacy.

Third, AE and the other exercise types form a complementary relationship between foundational prescriptions and precision prescriptions. AE, through broad-spectrum, low-dose activation across multiple pathways, accommodates a variety of pathological pathways and showed relatively favorable effects across all three outcomes and both substance subtypes, making it suitable as a reliable foundational prescription in scenarios with an unclear substance type, polysubstance use, multiple comorbid outcomes, or limited clinical resources; ME, MBE, and RSE, owing to their stronger mechanistic specificity, may serve as precision upgrade strategies targeting specific combinations in scenarios where the symptom target and substance type can be clearly defined. The practical implications of these findings are most directly applicable to SUD populations and rehabilitation settings similar to those represented in the included randomized controlled trials, particularly settings in which structured exercise interventions can be delivered and monitored. When applying these findings to populations differing substantially in age, sex composition, SUD severity, comorbidity profile, treatment setting, or cultural context, the symptom-targeted exercise recommendations should be adapted according to patient characteristics and local rehabilitation conditions (**Table 7**).

**Table 7:** Practical interpretation of exercise modalities based on the current network meta-analysis.

Exercise modality	Main signal in the current analysis	Practical interpretation
AE	Showed statistically supported benefits versus CON for addictive craving and negative affect, and ranked highest for the cognitive function outcome.	May be considered a foundational option when symptom targets are mixed or clinical resources are limited.
ME	Showed statistically supported benefits versus CON for addictive craving and negative affect, with the highest ranking for craving.	May be useful when craving or multiple symptom burdens are prominent.
MBE	Ranked favorably for craving in the StUD subgroup and showed a statistically supported effect versus CON in that subgroup.	May be considered when craving is accompanied by arousal, stress, or regulation-related symptoms.
RSE	Ranked relatively high for craving in the Non-StUD subgroup, although evidence remained less definitive.	May be considered as an adjunctive option where bodily control, motivation, or self-efficacy are relevant targets.
YME	Ranked relatively high for negative affect, but the confidence interval crossed zero.	May be considered an affect-oriented adjunct, while further confirmation is needed.

This table summarizes the main evidence signals and a cautious practical interpretation for each modality based on the current network meta-analysis; the interpretations are intended as supportive considerations (“may be considered”) rather than definitive treatment recommendations. AE, aerobic exercise; ME, multicomponent exercise; MBE, mind–body exercise; RSE, resistance exercise; YME, yoga- or mindfulness-based exercise; CON, control; StUD, stimulant use disorder; Non-StUD, non-stimulant use disorder.

## 5 Conclusion

The findings of this study indicate that the rehabilitation effects of different exercise interventions in patients with SUD show clear outcome specificity and substance-subtype-related differences in response. At the level of the three outcomes, compared with CON, ME and AE showed statistically supported benefits for addictive craving, and AE and ME showed statistically supported benefits for negative affect; YME ranked relatively high for negative affect but did not reach statistical significance; AE ranked highest for the cognitive-related outcome, although no intervention reached statistical significance versus CON for this outcome. At the substance-subtype level, ME, MBE, and AE showed statistically supported benefits for craving in patients with StUD, whereas AE showed a statistically supported benefit for craving in patients with Non-StUD. In addition, AE maintained relatively high rankings and a relatively stable effect direction across all three outcomes and both substance subgroups, and may serve as a foundational prescription option, whereas ME, MBE, and RSE may be more suitable as precision programs targeting specific symptom–substance combinations. These modality-specific and subgroup-specific interpretations should be regarded as evidence-informed but still requiring confirmation in larger, well-designed, multi-arm randomized trials. The present study argues that the optimization of SUD exercise-based rehabilitation should move beyond the simple question of “whether exercise is effective” and shift toward an evidence-based framework addressing “which exercise, for which patients, and targeting which symptoms.” The value of the “symptom target–substance type–exercise mechanism” framework proposed in this study lies in providing a basis for program selection in exercise interventions under different symptom burdens and substance subtypes, thereby helping move SUD exercise-based rehabilitation from empirical recommendation toward precision practice, and informing the design of subsequent multi-arm randomized controlled trials stratified by substance type, the optimization of exercise prescriptions, and clinical subgroup analyses.

## 6 Limitations and future research directions

First, the included studies differed somewhat in participant characteristics (including age, sex composition, SUD severity where reported, treatment setting, and cultural context), the main substance used, the form of exercise intervention, the intervention period, and the outcome measurement instruments. Although this study constructed separate networks by outcome type and assessed the stability of the results through consistency tests and sensitivity analyses, the clinical and methodological differences among studies may still have affected some of the effect estimates. In addition, because exercise protocols varied in intervention duration, findings from short-term or single-session protocols were interpreted according to their original post-intervention assessment context and were not overextended as evidence of long-term rehabilitation effects. Second, the number of studies in some exercise intervention nodes and subgroup networks was relatively limited, and the results yielded by different exercise modalities still require further study. In this context, potential small-study effects or publication bias cannot be fully excluded, particularly for exercise modalities supported by fewer trials. Future research should further strengthen the standardized design of the included exercise intervention programs, extend the follow-up period, and incorporate more clinically meaningful outcomes such as relapse rate, duration of maintained abstinence, recovery of social functioning, and neurobiological indicators, so as to advance the evidence on SUD exercise-based rehabilitation toward greater precision and translatability.

## Data Availability

The original contributions presented in the study are included in the article/supplementary material. Further inquiries can be directed to the corresponding authors.
